# NEGR1 deficiency disrupts lipid metabolism and steroidogenesis in Leydig cells, linking testosterone to behavior

**DOI:** 10.1016/j.jlr.2025.100892

**Published:** 2025-08-29

**Authors:** Poudel Rekha, Ara Yoo, Jangrae Kim, Soojin Lee

**Affiliations:** Department of Microbiology and Molecular Biology, Chungnam National University, Daejeon, Republic of Korea

**Keywords:** Cholesterol trafficking, steroid hormone, cholesterol/cells and tissues, neuronal growth regulator 1, testis, Leydig cell, testosterone

## Abstract

Neuronal growth regulator 1 (NEGR1) has been identified as a critical risk factor for major depressive disorders in humans. Although NEGR1 is predominantly expressed in the brain, its deletion in mice (*Negr1*^−/−^) results in abnormalities in peripheral tissues, suggesting a role beyond the nervous system, particularly in intracellular lipid trafficking. However, the role of NEGR1 in testosterone production has not yet been elucidated. Here, we demonstrate that *Negr1*^−/−^ mice exhibit significantly reduced serum and testicular testosterone levels, accompanied by diminished male reproductive behaviors. The expression of key testosterone-synthesizing enzymes was downregulated in Leydig cells, and histological analysis revealed disorganized testicular and epididymal structures with lipid droplet accumulation in testicular cells. Additionally, *Negr1*^−/−^ mice displayed a significant increase in abnormal sperm morphology. Notably, testosterone supplementation alleviated their impaired sexual behaviors and mitigated anxiety- and depression-like phenotypes. These findings highlight a crucial role for NEGR1 in testicular function, particularly in testosterone production and spermatogenesis, underscoring the intricate link between hormonal balance and mental health.

Leydig cells are male-specific cells that reside in the interstitial compartment of the testes and function as major sources of testosterone in mammalian males (1). Cholesterol serves as the primary precursor for testosterone synthesis, which is why Leydig cells are rich in lipid droplets and serve as a cholesterol reservoir (2). Leydig cells constitute approximately 2.7% of the total testicular volume, and an average rodent Leydig cell secretes approximately 0.5 ng of testosterone daily (3).

Testosterone production is regulated by luteinizing hormone, which stimulates testosterone biosynthesis by facilitating cholesterol mobilization and transport via the steroidogenic pathway (4). Steroidogenesis is a sequential process involving steroidogenic enzymes located in the mitochondria and the smooth endoplasmic reticulum (ER) (5). Testosterone is produced locally in the testes and diffuses into the seminiferous tubules, where it plays a crucial role in regulating spermatogenesis (6).

Defects in the development of adult Leydig cells can cause hypotestosteronemia, which impairs spermatogenesis and leads to the loss of normal germ cells (7). Decreased androgen levels can also cause histological changes in the testes as well as reduced sperm count and motility (8). Low testosterone not only affects reproductive function but can also lead to various sexual, somatic, and behavioral symptoms. This hormonal deficiency is associated with changes in body fat and muscle mass, contributing to obesity, atherosclerosis, decreased cognitive function, and increased depression and anxiety (9, 10). Additionally, elevated testosterone levels in aged male mice have been shown to reduce anxiety, improve affective performance, and enhance cognitive function (11).

Neuronal growth regulator 1 (NEGR1) is an extracellular membrane protein that contains three immunoglobulin-like domains (12). NEGR1 was originally discovered as a tumor-associated gene and was later recognized as a neuronal cell adhesion molecule that promotes neurite outgrowth and synapse assembly (13). Multiple genome-wide association studies (GWAS) have established NEGR1 as a significant locus associated with human pathologies, including obesity (14) and major depression (15, 16), and behavioral studies in Negr1 knockout (KO) mice reported increased anxiety- and depression-like traits (17). In contrast, we previously identified that *Negr1*^*−/−*^ mice had enlarged adipocytes with increased fat content (18) and that NEGR1 can modulate fatty acid transport in adipocytes by interacting with CD36 (cluster of differentiation 36) (19), suggesting that it has other roles in intracellular lipid trafficking (18, 19).

While we noticed that the breeding rate of *Negr1*^*−/−*^ mice is much lower than that of wild-type (WT) mice, a single-cell RNA sequencing study recognized NEGR1 as a molecular marker for Leydig cells in the human testis (20). Its high expression in Leydig cells was also observed in the single-cell RNA expression data from the Human Protein Atlas database (https://www.proteinatlas.org). However, the role of NEGR1 in testosterone production has not been elucidated. Therefore, we aimed to investigate the role of NEGR1 in testosterone biosynthesis and elucidate the anti-depressive effects of steroid hormones.

## Materials and methods

### Animals and cell culture

All experimental procedures were approved by the Institutional Animal Care and Use Committee of Chungnam National University (CNU IACUC), in accordance with national regulations and internationally recognized guidelines, including those of the American Veterinary Medical Association (AVMA), for the ethical use of laboratory animals. Both *Negr1*^*+/+*^ and *Negr1*^*−/−*^ C57BL6 mice were kept in 12 h light/dark cycles in a controlled environment at 22−24 °C and 55% humidity. The TM3 cells were maintained in Dulbecco’s modified Eagle’s medium (DMEM)/F12 (1:1) supplemented with 2.5% fetal bovine serum (Thermo Fisher Scientific) 5% horse serum (Thermo Fisher Scientific), and 15 mM HEPES.

### Western blot analysis

Cells and tissue samples were homogenized in radioimmunoprecipitation assay lysis buffer (Thermo Fisher Scientific) containing 1 mM phenylmethylsulfonyl fluoride and a protease inhibitor cocktail (MilliporeSigma, Burlington). Protein concentrations were determined using a bicinchonic acid protein assay kit (iNtRON Biotechnology Inc.). Immunoblotting analysis was performed as previously described [17]. Anti-NEGR1 antibodies were purchased from R&D Systems and Abcam, whereas anti-cytochrome P450 17A1 (Cyp17A1) antibody was purchased from GeneTex. Antibodies against 3β-hydroxysteroid dehydrogenase (3βHSD), steroidogenic acute regulatory protein (StAR), androgen receptor (AR), fatty acid binding protein 4 (FABP4), adipocyte differentiation-related protein (ADRP), glucose-regulated protein 78 (GRP78), and vinculin were obtained from Santa Cruz Biotechnology.

### RNA extraction and real-time quantitative polymerase chain reaction (RT-qPCR)

Total RNA was extracted from the testicular tissue using a Nucleospin RNA Extraction Kit (Macherey-Nagel), according to the manufacturer’s instructions. RT-qPCR was performed using the SYBR Green PCR Master MIX (Thermo Fisher Scientific). PCR was performed using the Bio-Rad CFX Connect Real-Time PCR Detection System (Bio-Rad). The relative fold change in gene expression was quantified using the 2^–ΔΔCt^ method, with β-actin serving as the internal control gene. The primer sequences used are listed in Supplemental Table S1.

### Histopathological analysis and lipid staining

Testicular and epididymal tissues were fixed overnight in 4% paraformaldehyde at 4 °C and embedded in paraffin for sectioning. Immunochemical analysis (IHC) was performed using the Vectastain Elite ABC Detection Kit (Vector Laboratories). For immunostaining, tissue sections were incubated with the appropriate primary antibodies in phosphate-buffered saline (PBS) for 2 h at room temperature, followed by incubation with fluorescent-linked secondary antibodies such as Alexa Fluor 488 anti-mouse IgG or Alexa Fluor 568 anti-goat IgG antibodies (Invitrogen) for 1 h. To visualize intracellular lipid droplets, tissue sections were stained with BODIPY 493/503 (2 μM, Thermo Fisher Scientific) for 10 min. For filipin staining, tissue sections were incubated with filipin (250 μg/ml in PBS) for 1 h. Imaging was performed on an Olympus BX51 microscope and analyzed using ImageJ software (National Institute of Health).

### Epididymal sperm parameter

Sperm motility and morphology were analyzed as previously described (21). Briefly, the caudal epididymis was collected and cut into pieces to facilitate the release of spermatozoa into prewarmed Ham’s F10 medium (Thermo Fisher Scientific). Sperm motility was assessed using an optical microscope at 100x magnification. After 10 random fields were examined, the average percentage of motile spermatozoa was calculated and recorded as total sperm motility. To observe sperm morphology, one drop of the sperm suspension was smeared on a glass slide and fixed using 4% paraformaldehyde. After air-drying, sections were stained with haematoxylin and eosin (H&E). The sperm abnormality rate was evaluated by observing at least 200 sperm per sample at 400x magnification. Sperm abnormalities included head, midpiece, and tail defects.

### Determination of testosterone and 17β-estradiol levels

Blood samples were collected by heart puncture from 12-week-old WT and *Negr1*^*−/−*^ mice (n = 7). Testosterone levels in the serum and testes were measured using a DetectX testosterone ELISA kit (K032-H; Arbor Assays) according to the manufacturer’s instructions. The testosterone concentration in the cell supernatant was measured using a testosterone ELISA kit (Uscn Life Science Inc). Plasma and testicular estrogen levels were determined using an ELISA kit for estradiol (E2) (Uscn Life Science Inc.).

### Isolation of primary Leydig cells

Primary Leydig cells were isolated as described previously (22). Briefly, testes obtained from 8−10-week-old mice were treated with collagenase (1 mg/ml) at 37 °C for 30 min. After the content settled for 5 min, the supernatant was filtered on a 45-μm cell strainer and centrifuged at 500 ×g for 5 min. The cells were washed twice and resuspended in DMEM/F12. The purity of Leydig cells was checked by staining with 3βHSD.

### Establishment of TM3-TetOne-NEGR1 cell line

To generate a tetracycline-inducible NEGR1 expression construct (pLVX-TetOne-NEGR1), the 3FLAG-NEGR1 fragment was amplified from the pcDNA4-3FLAG-NEGR1 plasmid (19). The PCR fragment was then assembled with the pLVX-TetOne backbone obtained from pLVX-TetOne-puro-GFP (#171123; Addgene) (23). Next, HEK293T cells were transfected with pLVX-TetOne-NEGR1 plasmids and psPAX2 and pMD2.G lentiviral packaging plasmids (kindly provided by Professor Jaeil Han, Chungnam National University) (24). The medium was changed 24 h post-transfection, and the culture supernatant was collected every 24 h for 2 days. Then, the TM3 Leydig cell line was transduced with the viral supernatant, and the cells were treated with 5 μg/ml puromycin (Invitrogen) for selection. To induce NEGR1 expression, cells were incubated with 1.5 μg/ml doxycycline (Sigma-Aldrich).

### Testosterone treatment

Healthy adult (8−12-week-old) male *Negr1*^*+/+*^ and *Negr1*^*−/−*^ mice were randomly assigned to four groups. Testosterone propionate (Tokyo Chemical Industry Co.) was dissolved in olive oil. The control group (n = 4) received an intraperitoneal injection of vehicle (olive oil), while the test groups (n = 5) received testosterone (25 mg/kg) once a day for 4 days. Behavioral tests were conducted 24 h after the final injection.

### Sexual and depression-like behavior test

Naive male and female adult (8−12-week-old) mice were used for the experiment. For individual behavior analysis, male mice were singly housed for 7 days, and their behavior was observed for 10 min in their home cage. Climbing activity was scored as male mice standing with both forepaws supported on the wall of the cage. Sniffing was defined as a male actively exploring a cage by sniffing. Male grooming was defined as self-grooming of the body, face, anogenital area, and trunk. Rearing was counted when males with both forepaws stood straight and stretched in the air.

Sexual behaviors were scored as previously described (25). For individual sexual behavior analysis, a female mouse was introduced into the home cage of a male mouse, and the behavior of the male mouse was video-recorded for 30 min. Anogenital sniffing was scored when the male mouse approached the female anogenital area by actively sniffing and touching. Mounting was counted when males using both forepaws climbed onto a female from behind for copulation. Intromission was scored as male pelvic thrust with a stable frequency for more than 6 s.

## Results

### Negr1-deficient male mice showed low sexual activities

**Fig. 1:**
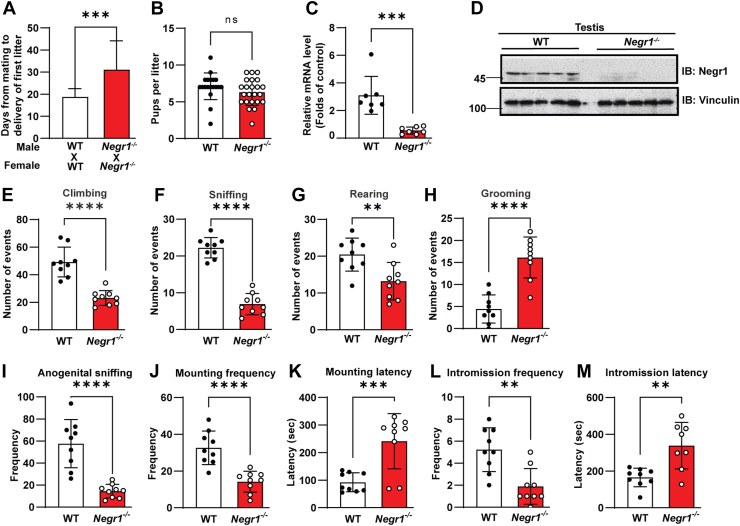
Deficits in male sexual behavior of *Negr1*^*−/−*^ mice. A: The time from breeding to the delivery of the first litter was measured following mating. B: The number of pups per litter was counted for both WT and *Negr1*^*−/−*^ mice. C: Quantitative RT-PCR was performed using total RNA isolated from adult testes. D: Immunoblotting analysis of testes lysates was conducted with an anti-NEGR1 antibody. (E–H) Male *Negr1*^+/+^ and *Negr1*^*−/−*^ mice (n = 9) were observed for individual behaviors, including climbing (E), sniffing (F), rearing (G), and grooming (H). (I–M) Male sexual behavior was assessed after introducing a female mouse into the cage. Behavioral tests included the frequency of anogenital behavior (I), frequency of mounting (J), latency to mount (K), frequency of intromission (L), and latency to intromission (M). ∗∗, *P* < 0.01; ∗∗∗, *P* < 0.001; ∗∗∗∗, *P* < 0.0001; ns, not significant.

As *Negr1*^*−/−*^ mice displayed subnormal fertility, we examined the duration from mating to delivery of the first litter (n = 21–25). *Negr1*^*−/−*^ mice required approximately 1.7-fold longer to obtain the first litter than did WT mice (Fig. 1A), although there was no significant difference in the number of pups (Fig. 1B). Based on that Negr1 is mildly expressed in several peripheral tissues including adipose tissue and skeletal muscle, in addition to the abundant expression in the brain (19), we examined the expression of Negr1 in testicular tissue. Both qRT-PCR (Fig. 1C) and immunoblotting (Fig. 1D) revealed that a considerable amount of Negr1 was expressed in the testes, which was completely abrogated in *Negr1*^*−/−*^ mice.

To evaluate the impact of Negr1 deficiency on male behaviors, we assessed mood and sexual behaviors. During a 10-minute test session, *Negr1*^*−/−*^ mice showed a decrease in climbing activity to ∼47% of that of control mice (Fig. 1E), indicating reduced explorative behavior. Sniffing activity and rearing frequency were also reduced in *Negr1*^*−/−*^ mice (Fig. 1F, G). Additionally, the self-grooming frequency significantly increased compared with that in WT mice (Fig. 1H), indicating an increased anxiety level in *Negr1*^*−/−*^ mice.

We then investigated the typical sexual behaviors of male mice and found that *Negr1*^*−/−*^ mice had an overall reduction in sexual aggression. *Negr1*^*−/−*^ mice exhibited a significant reduction in the frequency of anogenital sniffing of a female (Fig. 1I). The mounting frequency also decreased to ∼48% of that in WT mice (Fig. 1J), while it took approximately 2.6 times longer latency to mount (Fig. 1K). Furthermore, Negr1-deficient mice showed low intromission frequency (Fig. 1L) and required approximately 2-fold more time for the first intromission (Fig. 1M). Collectively, these results demonstrated that *Negr1*^*−/−*^ mice had decreased sexual motivation.

### Negr1 is highly expressed in the Leydig cells of the testis

**Fig. 2:**
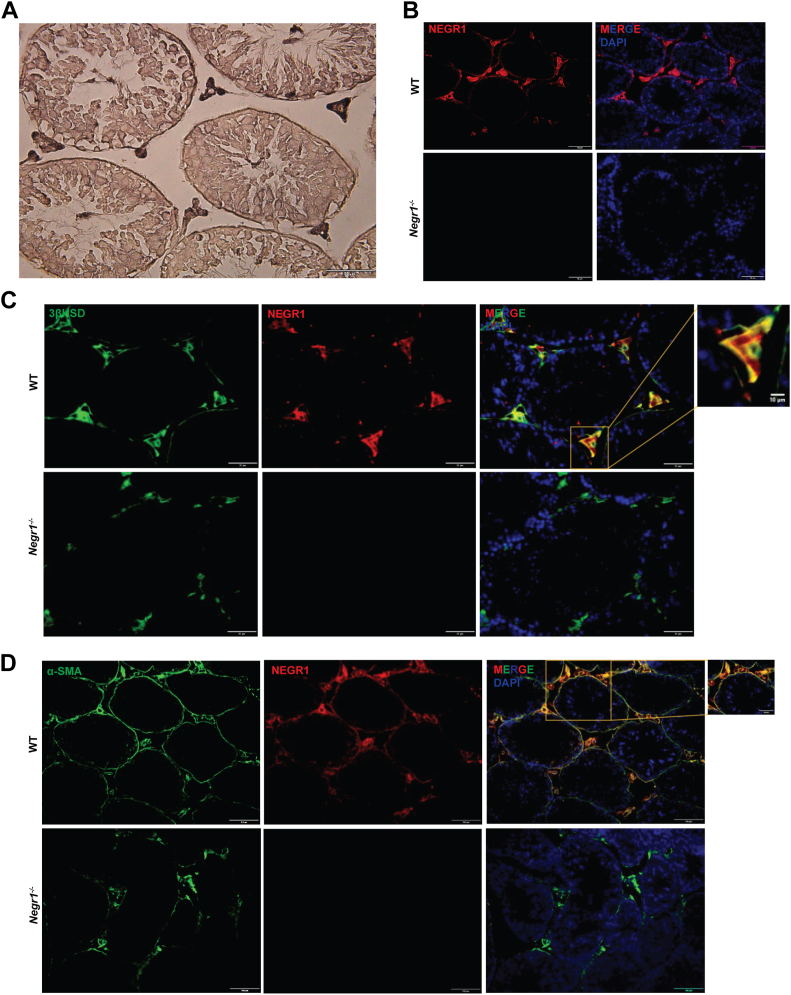
Expression of Negr1 in mouse testicular cells. A: Immunohistochemistry (IHC) was performed on paraffin-embedded testicular sections from WT mice using an anti-NEGR1 antibody. B: Immunofluorescence (IF) staining was carried out on testis tissue sections from both WT and *Negr1*^*−/−*^ mice. Sections were incubated with anti-NEGR1 antibody for 1 h, followed by incubation with Alexa Fluor 568 anti-goat IgG antibody (red). C: Co-immunofluorescence (Co-IF) was performed using a Leydig cell marker protein 3β-hydroxysteroid dehydrogenase (3βHSD). Sections were incubated with anti-NEGR1 (red) and anti-3βHSD (green) antibodies, followed by treatment with Alexa Fluor 488 anti-mouse IgG and Alexa Fluor 568 anti-goat IgG antibodies. D: Co-IF of testicular tissue sections were performed using an anti-α-smooth muscle actin (SMA) antibody to visualize peritubular myoid cells (green). Imaging was conducted using an Olympus BX51 microscope. Scale bar = 100 μm.

In order to determine the localization of Negr1 in the testes, we performed IHC with an anti-NEGR1 antibody using testicular sections from WT mice. We observed strong signals of Negr1 in the interstitial space, whereas moderate signals were observed in the boundary layer of seminiferous tubules (Fig. 2A). Similar expression patterns were observed in the immunofluorescence staining results (Fig. 2B), suggesting that Negr1 was expressed in Leydig cells and peritubular myoid cells of the testis. In contrast, considerable expression was not detected in Sertoli or germ cells.

To confirm these findings, we conducted co-immunofluorescence staining for specific cell markers. Testis tissue sections obtained from WT and *Negr1*^*−/−*^ mice were incubated with antibodies against NEGR1 and the Leydig cell marker, 3βHSD, an ER resident enzyme that plays a critical role in testosterone biosynthesis. As shown in Fig. 2C, the signals of 3βHSD were clearly co-localized with those of Negr1. To confirm this, co-immunostaining was performed using another Leydig cell marker, StAR, a mitochondrial cholesterol carrier (26). Negr1 was observed in the interstitial cells expressing StAR (Supplemental Fig. S1). Collectively, these data demonstrate the high expression of Negr1 in the Leydig cells of testes of adult mice.

To investigate whether Negr1 signals in the boundary zone of the seminiferous tubule were from the peritubular myoid cells, immunofluorescence staining was conducted using the peritubular myoid cell marker, α-smooth muscle actin (α-SMA). The α-SMA signals shown as a thin layer around seminiferous tubules well overlapped with Negr1 signals (Fig. 2D), implying that Negr1 is expressed in peritubular myoid cells.

As a control, co-immunostaining was also performed using the tissue sections of *Negr1*^*−/−*^ mice. In those slides, we noticed that the signals of 3βHSD, StAR, and α-SMA (lower panels of Figs. 2C, S1, and 2D, respectively) were weaker than those in WT mice. The stained cells were dispersed and highly disorganized. Considering that these marker proteins are essential for testicular function, this suggests that Negr1 deficiency may induce structural and functional alterations in the testis tissues.

### *Negr1*^*−/−*^ mice had low testosterone levels and highly disorganized testes

**Fig. 3:**
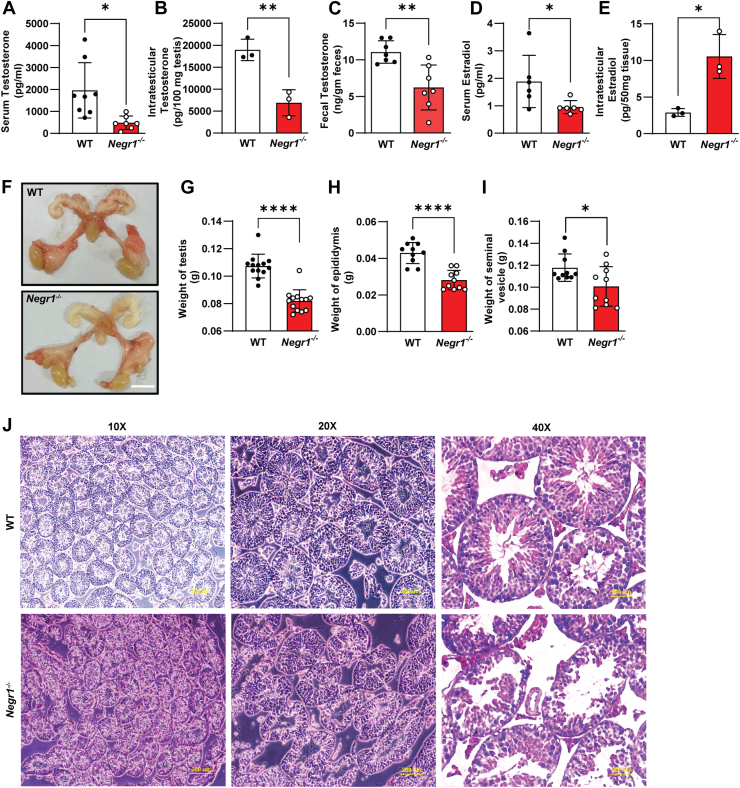
Negr1 KO mice exhibited low testosterone levels and disorganized testis. A–C: Testosterone levels were examined in the serum (A), testis (B), and feces (C) of 12-week-old WT and *Negr1*^*−/−*^ male mice (n = 7–8) using the DetectX Testosterone ELISA kit (Arbor Assays, Inc.) D–E: Estradiol concentrations in the serum (D) and testis (E) were measured with the Estradiol ELISA kit. F: Comparison of external gonadal appearance between WT and *Negr1*^*−/−*^ mice. (G–I) Weights of the testis (G), epididymis (H), and seminal vesicle (I) were measured using 10-week-old mice (n = 10–13). J: H&E staining of testicular sections from 12-week-old WT and *Negr1*^*−/−*^ mice. Scale bar = 200 μm. Data are presented as mean ± SE. ∗, *P* < 0.05; ∗∗, *P* < 0.01; ∗∗∗∗, *P* < 0.0001.

Based on the finding that Negr1 is highly expressed in Leydig cells, we determined testosterone levels in the serum, testis, and feces of 12-week-old *Negr1*^*−/−*^ mice. The testosterone levels of *Negr1*^*−/−*^ mice in all tested samples were significantly lower than those of WT (Fig. 3A–C), with the serum level of Negr1 KO mice being approximately 25% that of WT mice (Fig. 3A). Given that testosterone can be converted to estradiol, we also assessed estradiol levels. Estradiol concentration was significantly reduced in the serum of *Negr1*^*−/−*^ mice (Fig. 3D). However, the intratesticular estradiol level was highly increased (∼3.6-fold; Fig. 3E), suggesting that the production and secretion of steroid hormones were dysregulated in *Negr1*^*−/−*^ mice.

Next, we conducted morphological examinations of male reproductive organs and measured organ weights. Although there were no obvious morphological alterations, the overall color of *Negr1*^*−/−*^ mice was lighter than that of WT mice (Fig. 3F), possibly because of the high lipid content or low blood supply. In contrast, the weights of the testes, epididymis, and seminal vesicles in *Negr1*^*−/−*^ mice were significantly lower than those in WT mice (Fig. 3G–I), indicating that Negr1 deficiency negatively affected the development of the male sex organs.

We performed a histological examination using H&E staining of a testicular section from 12-week-old mice. Notably, the well-organized compact structure observed in WT mice was highly disrupted in *Negr1*^*−/−*^ mice (Fig. 3J). The degenerative features of *Negr1*^*−/−*^ mice include unevenly contracted seminiferous tubule epithelium, depletion of germ cells, spermatocytes, and spermatids, and a reduction in the number of cells within the adluminal compartment. Additionally, the distribution of interstitial cells was irregular and sparse. When we examined 5-week-old mice, no significant differences were observed in organ weights or histological morphology between WT and *Negr1*^*−/−*^ mice (Supplemental Fig. S2), indicating that the effect of Negr1 on the male reproductive organs becomes more evident as age increases.

### Decreased expression of testicular steroidogenic enzymes in *Negr1*^*−/−*^ mice

**Fig. 4:**
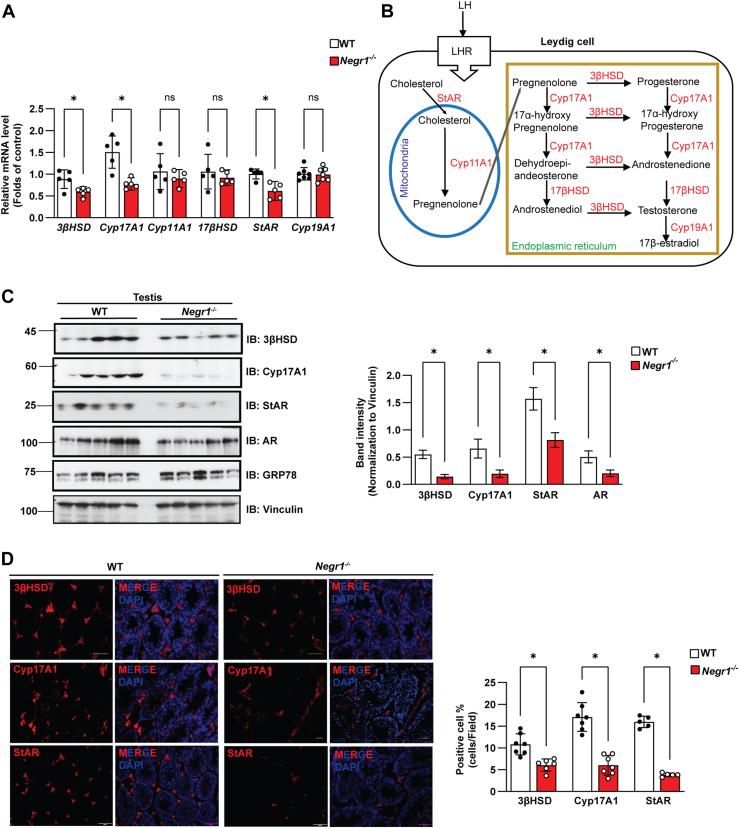
**Expression levels of steroidogenic enzymes in the testis of Negr1 KO mice**. A: Quantitative RT-PCR analysis of mRNA levels for steroidogenic enzymes in the testes of WT and *Negr1*^*−/−*^ mice (n = 5). ∗, *P* < 0.05. B: Schematic of testosterone and 17β-estradiol biosynthetic pathways in Leydig cells. C: Immunoblot analysis of testosterone-producing enzymes in testis lysates (n = 5), with protein levels normalized to vinculin. D: Immunostaining of testicular sections from 12-week-old mice using antibodies against 3βHSD, Cyp17A1, and StAR. Positive cells were counted using Image J. ∗, *P* < 0.05. Cyp17A1, cytochrome P450 17A1; StAR, steroidogenic acute regulatory protein; AR, androgen receptor; GRP78, glucose-regulated protein 78.

Considering that the plasma and testicular testosterone levels were significantly reduced in *Negr1*^*−/−*^ mice, we investigated whether the expressions of key enzymes involved in testosterone production were altered. We performed qRT-PCR using total RNA extracted from the testes of WT and Negr1 KO mice (n = 3–7). Among the major enzymes involved in steroidogenesis, the mRNA levels of 3βHSD, StAR, and Cyp17A1 were decreased in Negr1 KO mice, whereas no significant change was detected with Cyp11, Cyp19, and 17βHSD (Fig. 4A, B). Subsequent immunoblotting confirmed the lowered protein levels of 3βHSD, StAR, Cyp17A1, and AR in the testes of Negr1-deficient mice (Fig. 4C).

To further examine the previous findings, we stained testis tissue sections with specific antibodies against 3βHSD, StAR, and Cyp17A1. The overall signal intensities of these three enzymes in *Negr1*^*−/−*^ mice were weaker than those in WT mice, as verified by positive cell quantification (Fig. 4D). Overall, several key enzymes responsible for testosterone biosynthesis were downregulated in the Negr1-deficient mice.

### Loss of Negr1 causes defects in spermatogenesis

**Fig. 5:**
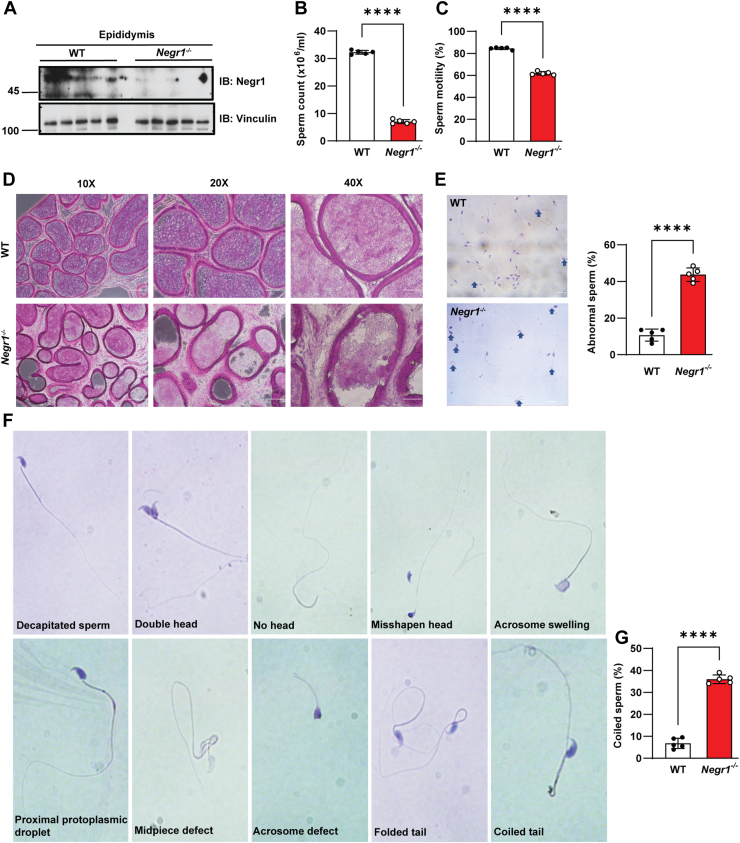
Evaluation of sperm parameters in Negr1 KO mice. A: Negr1 expression in the epididymis was examined by immunoblotting. B: Viable spermatozoa from the cauda epididymis (n = 5) were counted using trypan blue staining. C: Sperm motility was assessed by calculating the percentage of motile sperm across 10 random fields. D: H&E staining of caudal epididymis sections from WT and *Negr1*^*−/−*^ mice. E: Sperm morphology was examined after H&E staining, with arrows indicating abnormal sperm. Percentages were calculated from five independent experiments. Scale bars = 50 μm. F: Enlarged images of deformed sperm observed in *Negr1*^*−/−*^ mice. G: The percentage of coil-tailed sperm was quantified. Data represent mean ± SE. ∗∗∗∗, *P* < 0.0001.

As *Negr1*^*−/−*^ mice showed an enlarged epididymal fat mass (18), we examined whether Negr1 was expressed in the epididymis and observed a weak Negr1 band by immunoblotting (Fig. 5A). Semen samples were collected from the caudal epididymis, and sperm cells were stained with trypan blue to label dead cells. There was a marked decline in the sperm count of *Negr1*^*−/−*^ mice to only approximately 22% of that of the WT control (Fig. 5B). Sperm motility of *Negr1*^*−/−*^ mice was also significantly lower than that of WT mice (Fig. 5C), indicating that both sperm number and quality declined with Negr1 deficiency.

Next, we performed H&E staining of epididymal tissue sections. As shown in Fig. 5D, the epididymis of *Negr1*^*−/−*^ mice showed substantial morphological disruption of the epithelial structure, with a marked decrease in the number of sperm within the lumen. We then evaluated sperm morphology after H&E staining and observed a higher proportion of abnormal sperms in *Negr1*^*−/−*^ mice (Fig. 5E). Various morphological defects were observed, such as decapitated sperms, double heads, no head, misshapen head, acrosome swelling, proximal protoplasmic droplets, mid-piece defects, and folded and coiled tails (Fig. 5F). Coiled and folded tails were prevalent among the identified abnormalities (Fig. 5G). These findings indicated that Negr1 deficiency adversely affected sperm production, motility, and morphology.

### Impaired cholesterol and lipid trafficking in testicular cells

**Fig. 6:**
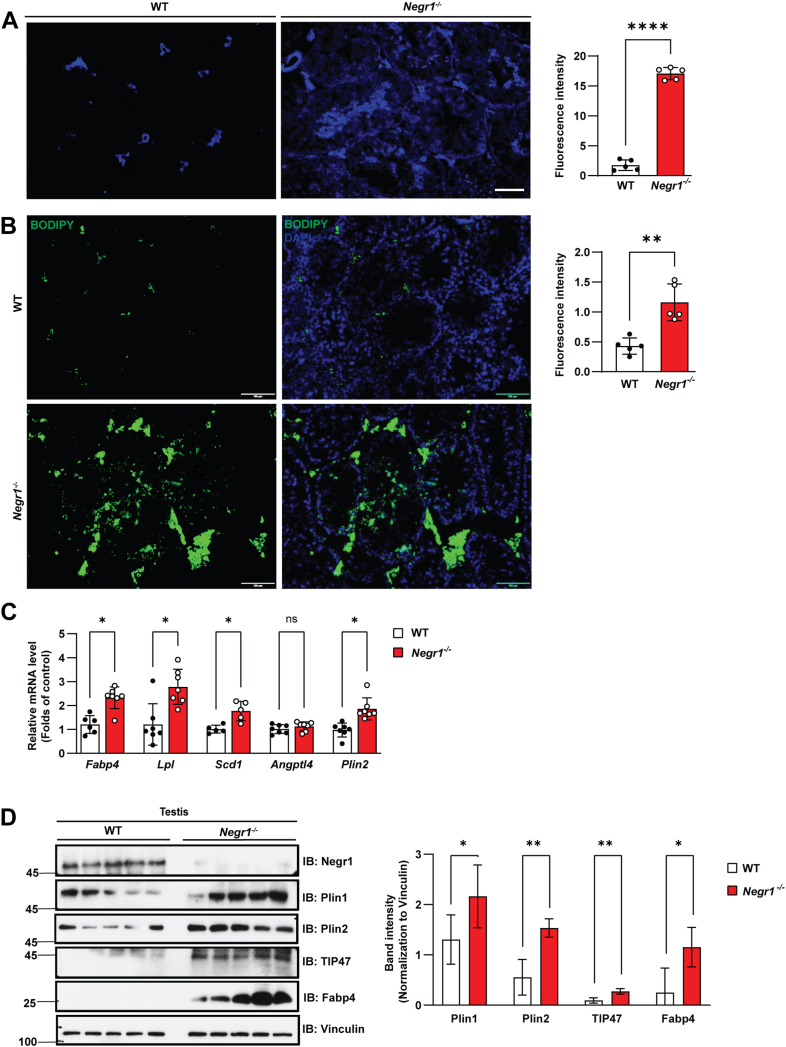
Increased lipid accumulation in testicular cells of *Negr1*^*−/−*^ mice. A: Testis sections from 12-week-old WT and *Negr1*^*−/−*^ mice were stained with filipin for 1 h to detect cholesterol accumulation. Fluorescence intensity was measured with Image J. ∗∗∗∗, *P* < 0.0001. B: Intracellular lipid droplets were visualized using BODIPY 493/503 staining. ∗∗, *P* < 0.01. C: Quantitative RT-PCR analysis of lipid metabolism-related genes in the testes (n = 7). D: Expression of lipid storage marker proteins was assessed by immunoblotting of total testis lysates. Fabp4, fatty acid binding protein 4; Lpl, lipoprotein lipase; Scd1, stearoyl-coA saturase 1; Angptl4, angiopoietin-like 4; Plin1−2, perilipin 1 and 2; TIP47, tail-interacting protein of 47 kDa.

Based on that Negr1 KO mice had increased intracellular lipid deposits in peripheral tissues, including adipose tissues and the liver (18), we evaluated whether NEGR1 is also involved in lipid metabolism in the testes. We performed filipin staining using testis tissue blocks and observed that cholesterol levels in Negr1-deificient cells were higher than those in WT controls (Fig. 6A). We also conducted BODIPY staining to visualize the neutral lipids. The fluorescence intensity was higher in *Negr1*^*−/−*^ mice than in WT mice (Fig. 6B). Notably, in both filipin and BODIPY staining, the fluorescence signals were observed only in the interstitial Leydig cells of WT mice. However, the positive signals of *Negr1*^*−/−*^ mice were detected more widely, even inside the seminiferous tubules.

Next, we examined the mRNA expression of the genes involved in lipid metabolism using qRT-PCR. Compared to WT mice, Negr1-depleted testicular cells showed higher expression of adipocyte marker genes, including fatty acid binding protein 4 (Fabp4), lipoprotein lipase (Lpl), stearoyl-CoA desaturase 1 (Scd1), and perilipin-2 (Plin2) (Fig. 6C). Immunoblot analysis further revealed that protein levels of Plin1, Plin2, Plin3/TIP47 (tail-interacting protein of 47 kDa), and Fabp4 were markedly increased in *Negr1*^*−/−*^ mice (Fig. 6D). Consistently, immunostaining of testis tissue sections confirmed elevated expression of Plin1, Plin2, and TIP47 in the absence of Negr1 (Supplemental Fig. S3). Collectively, these findings indicate that Negr1 deficiency profoundly impaired intracellular lipid trafficking in testicular cells.

### Exogenous testosterone increased the mating behavior of *Negr1*^*−/−*^ mice

**Fig. 7:**
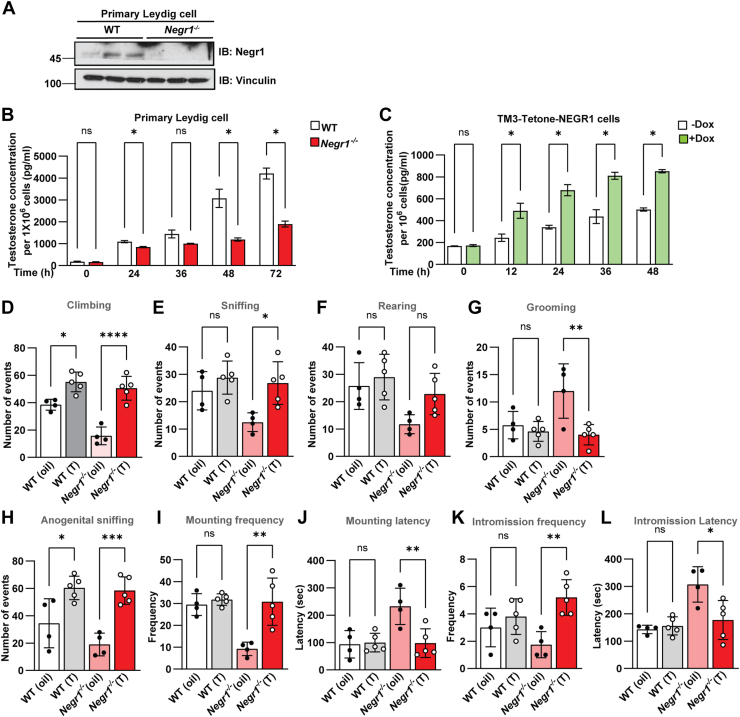
Exogenous testosterone modulated sexual and depression-like behaviors in *Negr1*^*−/−*^ mice. A: Negr1 expression in primary Leydig cells from WT and *Negr1*^*−/−*^ mice. B: Testosterone concentrations in culture supernatants from Leydig cells were measured. C: TM3-TetOne-NEGR1 cells were treated with doxycycline for 24 h to induce NEGR1 expression, and testosterone secretion was normalized to cell number. D–L: WT and *Negr1*^*−/−*^ male mice (8−12 weeks old) were randomly grouped (n = 4–5/group) and injected with vehicle (olive oil) or testosterone for 4 days. Behaviors were monitored: climbing (D), sniffing (E), rearing (F), and grooming (E) for 10 min, and male sexual behavior was observed for 30 min after introducing a female mouse: anogenital sniffing frequency (H), mounting frequency (I), mounting latency (J), intromission frequency (K), and intromission latency (L). Data represent mean ± SE. ∗, *P* < 0.05; ∗∗, *P* < 0.01; ∗∗∗, *P* < 0.001.

To investigate whether Negr1 expression affects testosterone secretion, we isolated primary Leydig cells from 8-week-old WT and *Negr1*^*−/−*^ mice (Fig. 7A). Culture supernatants were collected at different time points (24, 36, 48, and 72 h) after changing the media. Although the concentration of testosterone in the culture media gradually increased in both cell types, the secreted testosterone levels markedly reduced in Negr1-deficient Leydig cells (Fig. 7B). Next, we generated pLVX-Tetone-FLAG-NEGR1, a lentiviral vector for inducible expression of NEGR1, and established an inducible NEGR1-overexpressing cell line generated using the TM3 Leydig cell line (TM3-TetOne-NEGR1). Secreted testosterone levels in TM3-TetOne-NEGR1cells were elevated upon induction with doxycycline compared to those in uninduced cells (Fig. 7C). These data demonstrate that NEGR1 expression is closely related to testosterone release in Leydig cells.

Finally, we examined whether exogenous testosterone could reverse the phenotypical and biochemical alterations observed in the testes of *Negr1*^*−/−*^ mice, as well as their behavioral deficits. Both *Negr1*^*+/+*^ and *Negr1*^*−/−*^ male mice were randomly divided into two groups, with one group (n = 5) receiving daily intraperitoneal injections of testosterone for 4 days. Testosterone treatment did not induce significant changes in overall testicular morphology or in the protein expression of key steroidogenic enzymes (Supplemental Fig. S4). However, testosterone supplementation markedly reduced intracellular lipid content and decreased the abundance of lipid droplet-associated proteins, particularly Plin1 and Plin2. These findings suggest that exogenous testosterone promotes lipolysis and alleviates lipid accumulation in the testes of *Negr1*^*−/−*^ mice.

The impact of testosterone treatment on most behaviors of WT mice was not significant, except for climbing and anogenital sniffing behaviors, which were elevated by testosterone injection. In contrast, the behavior of *Negr1*^*−/−*^ mice was profoundly affected by the administration of testosterone compared with oil-injected controls. The frequencies of explorative behaviors (climbing and sniffing) in Negr1-deficient mice were increased by exogenous testosterone to almost the same level as in WT mice (Fig. 7D, E). Depression- and anxiety-like behaviors (grooming and rearing) also considerably improved after testosterone injection (Fig. 7F, G).

Regarding sexual behavior, the reduced anogenital sniffing and mounting behaviors in *Negr1*^*−/−*^ mice were significantly restored by testosterone treatment (Fig. 7H, I). Additionally, the mounting latency was shortened to almost the same level as that in WT mice (Fig. 7J), indicating sexual aggression toward female mice. Furthermore, exogenous testosterone increased intromission frequency and decreased intromission latency of *Negr1*^*−/−*^ mice (Fig. 7K, L). Altogether, these findings indicate that testosterone treatment is effective in alleviating depressive symptoms and sexual activity of *Negr1*^*−/−*^ mice.

## Discussion

In this study, we investigated the role of NEGR1 in the development and function of the Leydig cells of the testes. We found that NEGR1 is predominantly expressed in Leydig cells and NEGR1 depletion significantly influences steroidogenesis and spermatogenesis in the testes. To our knowledge, this is the first report to elucidate the role of NEGR1 in testosterone production, linking its deficiency to both biochemical and morphological abnormalities in the male reproductive system, and potentially to the psychiatric symptoms observed in *Negr1*^*−/−*^ mice.

Steroidogenesis is a complex process in which cholesterol is converted into steroid hormones. Free cholesterol in the intracellular membrane—distinct from cholesterol tightly bound to the plasma membrane—is the true substrate for steroidogenesis (5). Two Niemann−Pick type C (NPC) proteins, NPC1 and NPC2, are the only known essential factors for endosomal trafficking of cholesterol (5). NPC disease, caused by autosomal mutations in NPC1 or NPC2, is characterized by progressive neurological symptoms and is often accompanied by psychiatric manifestations such as psychosis and affective disorders (27). NPC2 is a small lysosomal protein that facilitates cholesterol transfer from endolysosomes to the plasma membrane (28). Our previous study revealed that NEGR1 interacts with NPC2 and contributes to intracellular cholesterol transport (29).

As NEGR1 is highly expressed in the cerebral cortex and hippocampus of the adult brain, its neuronal function became a subject of interest when a GWAS discovered NEGR1 as a locus associated with human obesity (14). However, the findings that *Negr1*^*−/−*^ mice show abnormalities in peripheral tissues such as increased adiposity, hepatic fat accumulation, and muscle atrophy (18), and NEGR1 interacts with lipid carriers in adipocytes (19), support the concept that NEGR1 has non-neuronal functions in lipid transport and metabolism in peripheral organs. Here, we show that NEGR1 deficiency causes pronounced lipid droplet accumulation in testicular cells, reduced expression of steroidogenic enzymes, and markedly decreased serum and testicular testosterone levels (Figs. 3 and 4). The elevated expression of genes related to lipid uptake and storage (Fig. 6C, D) further supports the conclusion that NEGR1 is essential for efficient cholesterol mobilization in the testes. Among testicular cells, Leydig cells have a particularly high demand for intracellular cholesterol to sustain testosterone synthesis, and appear especially vulnerable to this defect. NEGR1 loss likely leads to serious stagnation of free endosomal cholesterol—the genuine precursor of steroids—ultimately impairing steroidogenesis and spermatogenesis.

Estrogens are synthesized from aromatizable androgens such as testosterone by CYP19A1/aromatase (Fig. 4B), which mainly occurs in male Leydig cells (30). Ironically, the testicular estrogen level of Negr1-deficient mice was several folds higher than that of WT mice despite low plasma estrogen levels (Fig. 3D, E); this finding was notable because the CYP19A1 level was not altered in testes of *Negr1*^*−/−*^ mice (Fig. 4A). As CYP19A1 is an ER-resident enzyme, estrogen biosynthesis may directly depend on the local testosterone concentration inside the ER. We speculated that impaired lipid transport in *Negr1*^*−/−*^ mice could alter compartment-specific testosterone availability, thereby increasing local estrogen synthesis. Given that testosterone concentration in the testes of adult male mice is at least two orders of magnitude greater than estrogen (31) (see also Fig. 3B, E), even modest shifts could substantially affect estrogen concentrations.

Testosterone is also required for the initiation and maintenance of spermatogenesis (32). Sertoli cells are major transducers of testosterone signaling required for germ cell survival and development (33). Testicular testosterone concentration is approximately 10-fold higher than the serum level in rats and 25- to 125-fold higher in men (7). In the absence of relatively high levels of testosterone, germ cells fail to complete their maturation, resulting in low or no sperm (7). Therefore, the several-fold decrease in testicular testosterone levels in *Negr1*^*−/−*^ mice seems to be a direct cause of the demolished organ structure (Fig. 3J) and decreased sperm production (Fig. 5B). This low testosterone level may also explain the increased morphological abnormalities in sperm (Fig. 5F) and reduced fertility (Fig. 1A).

Low testosterone levels are associated with mood disorders (34, 35), and in this study, testosterone supplementation in *Negr1*^*−/−*^ mice restored sexual behaviors and partially ameliorated anxiety- and depression-like phenotypes (Fig. 7D–L). These data suggest that the behavioral changes may arise, at least in part, as downstream effects of impaired testosterone production. Given the established relationship between hypogonadism, infertility, and mood disorders, our findings support a mechanistic link between reproductive lipid metabolism and mental health.

Steroid hormones are important modulators of neurogenesis (36). Testosterone enhances hippocampal neurogenesis and synaptic function (36, 37). Estrogen can modulate adult neurogenesis by altering neurotransmitters (38). It has been previously reported that *Negr1*^*−/−*^ mice have defects in adult hippocampal neurogenesis (17). Thus, although the precise mechanism remains to be clarified, low testosterone levels in Negr1-deficient mice may impair adult neurogenesis and neuronal homeostasis, contributing to the development of depression-like behaviors.

In conclusion, this study identifies NEGR1 as a previously unrecognized regulator of cholesterol trafficking and testosterone biosynthesis in Leydig cells. By impairing lipid mobilization, NEGR1 deficiency disrupts steroid hormone production, spermatogenesis, and testicular architecture, with secondary systemic effects on reproductive and affective behaviors. These results expand NEGR1’s functional significance beyond the nervous system, highlighting its broader role in lipid metabolism within peripheral tissues and its potential relevance to disorders linking metabolic, reproductive, and neuropsychiatric domains.

## Data availability

The data presented in this study are available from the corresponding author upon reasonable request.

## Supplemental data

This article contains supplemental data.

## Conflict of interest

The authors declare no conflicts of interest related to the content of this article.

## References

[1] Bhattacharya I., Dey S. (2022). Emerging concepts on leydig cell development in fetal and adult testis. Front. Endocrinol. (Lausanne).

[2] Wang W., Wei S., Li L., Su X., Du C., Li F. (2015). Proteomic analysis of murine testes lipid droplets. Sci. Rep..

[3] Mori H., Christensen A.K. (1980). Morphometric analysis of leydig cells in the normal rat testis. J. Cell Biol..

[4] Hu J., Zhang Z., Shen W.J., Azhar S. (2010). Cellular cholesterol delivery, intracellular processing and utilization for biosynthesis of steroid hormones. Nutr. Metab. (Lond).

[5] Miller W.L., Bose H.S. (2011). Early steps in steroidogenesis: intracellular cholesterol trafficking. J. Lipid Res..

[6] Smith L.B., Walker W.H. (2014). The regulation of spermatogenesis by androgens. Semin. Cell Dev. Biol..

[7] Walker W.H. (2011). Testosterone signaling and the regulation of spermatogenesis. Spermatogenesis.

[8] Semet M., Paci M., Saias-Magnan J., Metzler-Guillemain C., Boissier R., Lejeune H. (2017). The impact of drugs on male fertility: a review. Andrology.

[9] Steinfeld K., Beyer D., Muhlfeld C., Mietens A., Eichner G., Altinkilic B. (2018). Low testosterone in ApoE/LDL receptor double-knockout mice is associated with rarefied testicular capillaries together with fewer and smaller leydig cells. Sci. Rep..

[10] Rohr U.D. (2002). The impact of testosterone imbalance on depression and women's health. Maturitas.

[11] Frye C.A., Edinger K., Sumida K. (2008). Androgen administration to aged male mice increases anti-anxiety behavior and enhances cognitive performance. Neuropsychopharmacology.

[12] Kim H., Hwang J.S., Lee B., Hong J., Lee S. (2014). Newly identified cancer-associated role of human neuronal growth regulator 1 (NEGR1). J. Cancer.

[13] Noh K., Park J.C., Han J.S., Lee S.J. (2020). From bound cells comes a sound mind: the role of neuronal growth regulator 1 in psychiatric disorders. Exp. Neurobiol..

[14] Willer C.J., Speliotes E.K., Loos R.J., Li S., Lindgren C.M., Heid I.M. (2009). Six new loci associated with body mass index highlight a neuronal influence on body weight regulation. Nat. Genet..

[15] Howard D.M., Adams M.J., Shirali M., Clarke T.K., Marioni R.E., Davies G. (2018). Genome-wide association study of depression phenotypes in UK biobank identifies variants in excitatory synaptic pathways. Nat. Commun..

[16] Deng Y.T., Ou Y.N., Wu B.S., Yang Y.X., Jiang Y., Huang Y.Y. (2022). Identifying causal genes for depression via integration of the proteome and transcriptome from brain and blood. Mol. Psychiatry.

[17] Noh K., Lee H., Choi T.Y., Joo Y., Kim S.J., Kim H. (2019). Negr1 controls adult hippocampal neurogenesis and affective behaviors. Mol. Psychiatry.

[18] Joo Y., Kim H., Lee S., Lee S. (2019). Neuronal growth regulator 1-deficient mice show increased adiposity and decreased muscle mass. Int. J. Obes. (Lond).

[19] Yoo A., Joo Y., Cheon Y., Lee S.J., Lee S. (2022). Neuronal growth regulator 1 promotes adipocyte lipid trafficking via interaction with CD36. J. Lipid Res..

[20] Nie X., Munyoki S.K., Sukhwani M., Schmid N., Missel A., Emery B.R. (2022). Single-cell analysis of human testis aging and correlation with elevated body mass index. Dev. Cell.

[21] Suleiman J.B., Abu Bakar A.B., Noor M.M., Nna V.U., Othman Z.A., Zakaria Z. (2021). Bee bread mitigates downregulation of steroidogenic genes, decreased spermatogenesis, and epididymal oxidative stress in male rats fed with high-fat diet. Am. J. Physiol. Endocrinol. Metab..

[22] Wang J.Q., Cao W.G. (2016). Morphological characterization of adult mouse leydig cells in culture. Biochem. Biophys. Res. Commun..

[23] Lukow D.A., Sausville E.L., Suri P., Chunduri N.K., Wieland A., Leu J. (2021). Chromosomal instability accelerates the evolution of resistance to anti-cancer therapies. Dev. Cell.

[24] Manjunath H., Zhang H., Rehfeld F., Han J., Chang T.C., Mendell J.T. (2019). Suppression of ribosomal pausing by eIF5A is necessary to maintain the fidelity of start codon selection. Cell Rep..

[25] Haga S., Hattori T., Sato T., Sato K., Matsuda S., Kobayakawa R. (2010). The male mouse pheromone ESP1 enhances female sexual receptive behaviour through a specific vomeronasal receptor. Nature.

[26] Payne A.H., Hales D.B. (2004). Overview of steroidogenic enzymes in the pathway from cholesterol to active steroid hormones. Endocr. Rev..

[27] Rego T., Farrand S., Goh A.M.Y., Eratne D., Kelso W., Mangelsdorf S. (2019). Psychiatric and cognitive symptoms associated with Niemann-Pick type C disease: neurobiology and management. CNS Drugs.

[28] Juhl A.D., Lund F.W., Jensen M.L.V., Szomek M., Heegaard C.W., Guttmann P. (2021). Niemann Pick C2 protein enables cholesterol transfer from endo-lysosomes to the plasma membrane for efflux by shedding of extracellular vesicles. Chem. Phys. Lipids.

[29] Kim H., Chun Y., Che L., Kim J., Lee S., Lee S. (2017). The new obesity-associated protein, neuronal growth regulator 1 (NEGR1), is implicated in Niemann-Pick disease type C (NPC2)-mediated cholesterol trafficking. Biochem. Biophys. Res. Commun..

[30] Hess R.A. (2003). Estrogen in the adult male reproductive tract: a review. Reprod. Biol. Endocrinol..

[31] Cooke P.S., Nanjappa M.K., Ko C., Prins G.S., Hess R.A. (2017). Estrogens in Male physiology. Physiol. Rev..

[32] Grande G., Barrachina F., Soler-Ventura A., Jodar M., Mancini F., Marana R. (2022). The role of testosterone in spermatogenesis: lessons from proteome profiling of human spermatozoa in testosterone deficiency. Front. Endocrinol. (Lausanne).

[33] Toocheck C., Clister T., Shupe J., Crum C., Ravindranathan P., Lee T.K. (2016). Mouse spermatogenesis requires classical and nonclassical testosterone signaling. Biol. Reprod..

[34] Zito S., Nosari G., Pigoni A., Moltrasio C., Delvecchio G. (2023). Association between testosterone levels and mood disorders: a minireview. J. Affect. Disord..

[35] Kiani Z., Fakari F.R., Hakimzadeh A., Hajian S., Fakari F.R., Nasiri M. (2023). Prevalence of depression in infertile men: a systematic review and meta-analysis. BMC Public Health.

[36] Wainwright S.R., Galea L.A. (2013). The neural plasticity theory of depression: assessing the roles of adult neurogenesis and PSA-NCAM within the hippocampus. Neural Plast..

[37] Zhang Y., Chen M., Chen H., Mi S., Wang C., Zuo H. (2024). Testosterone reduces hippocampal synaptic damage in an androgen receptor-independent manner. J. Endocrinol..

[38] Garcia-Segura L.M., Azcoitia I., DonCarlos L.L. (2001). Neuroprotection by estradiol. Prog. Neurobiol..

