# Supplementary material for: NEGR1 deficiency disrupts lipid metabolism and steroidogenesis in Leydig cells, linking testosterone to behavior
Source: J Lipid Res. 2025 Aug 29;66(10):100892. doi: 10.1016/j.jlr.2025.100892 (PMC12516559; doi:10.1016/j.jlr.2025.100892)
Supplement: Supplementary Materials [file mmc1.docx]

Title: NEGR1 deficiency disrupts lipid metabolism and steroidogenesis in Leydig cells, linking testosterone to behavior

Authors: Poudel Rekha, Ara Yoo, Jangrae Kim, and Soojin Lee^,*^

^*^Correspondence: Soojin Lee ([leesoojin@cnu.ac.kr](mailto:leesoojin@cnu.ac.kr))

**Supplementary Table S1** Primers for quantitative-RT-PCR.

| **Name** | **Sequence (5′ -> 3′)** |
| --- | --- |
| StAR | Forward: TAAACTCACTTGGCTGCTCAGTATTG  Reverse: GGTGGTTGGCGAACTCTATCTG |
| 3βHSD | Forward: AGCTCTGGACAAAGTATTCCGA  Reverse: GCCTCCAATAGGTTCTGGGT |
| 17βHSD | Forward: AGTTGGCCAGACATGGACTC  Reverse: ACACAGCTTCCAGTGGTCCTC |
| Cyp17A1 | Forward: GAGGTGAAGAGGAAGATCCAAA  Reverse: ATACGAAGCACTTCTCGGATAG |
| Cyp19A1 | Forward: CGGGCTACGTGGATGTGTT  Reverse: GAGCTTGCCAGGCGTTAAAG |
| β-Actin | Forward: GGACTCCTATGTGGGTGACG  Reverse: CTTCTCCATGTCGTCCCAGT |
| CYP11A1 | Forward: AAGACCTGGAAGGACCATGC  Reverse: CACCAGGGTACTGGCTGAAG |
| Fabp4 | Forward: ACAGCTCCTCCTCGAAGGTTTAC  Reverse: TCCTGTCGTCTGCGGTGATT |
| Lpl | Forward: CGTAGCAGGAAGTCTGACCAATAAG  Reverse: TGCAATCACACGGATGGCTT |
| Plin2 | Forward: GCTGGAGCCAAGGATTCTGT  Reverse: TCATGAACTGCACCATCCCC |
| Angptl4 | Forward: CAGCAGCAGAGATACCTATCAAAGC  Reverse: CCTGTGTAAGTGGGTGGCGTT |
| Scd1 | Forward: ACCTGCCTCTTCGGGATTTTC  Reverse: GGCCCATTCGTACACGTCATT |

StAR, Steroidogenic acute regulatory protein; 3βHSD, 3β-hydroxysteroid dehydrogenase; 17βHSD, 17β-hydroxysteroid dehydrogenase; Cyp17A1, Cytochrome P450 17A1; Cyp19A1, Cytochrome P450 19A1; Cyp11A1, Cytochrome P450 11A1; Fabp4, Fatty acid binding protein 4; Lpl, lipoprotein lipase; Plin2, Perilipin 2; Angptl4, Angiopoietin-like 4; Scd1, Stearoyl-CoA desaturase.

**Supplementary Figure S1**

**
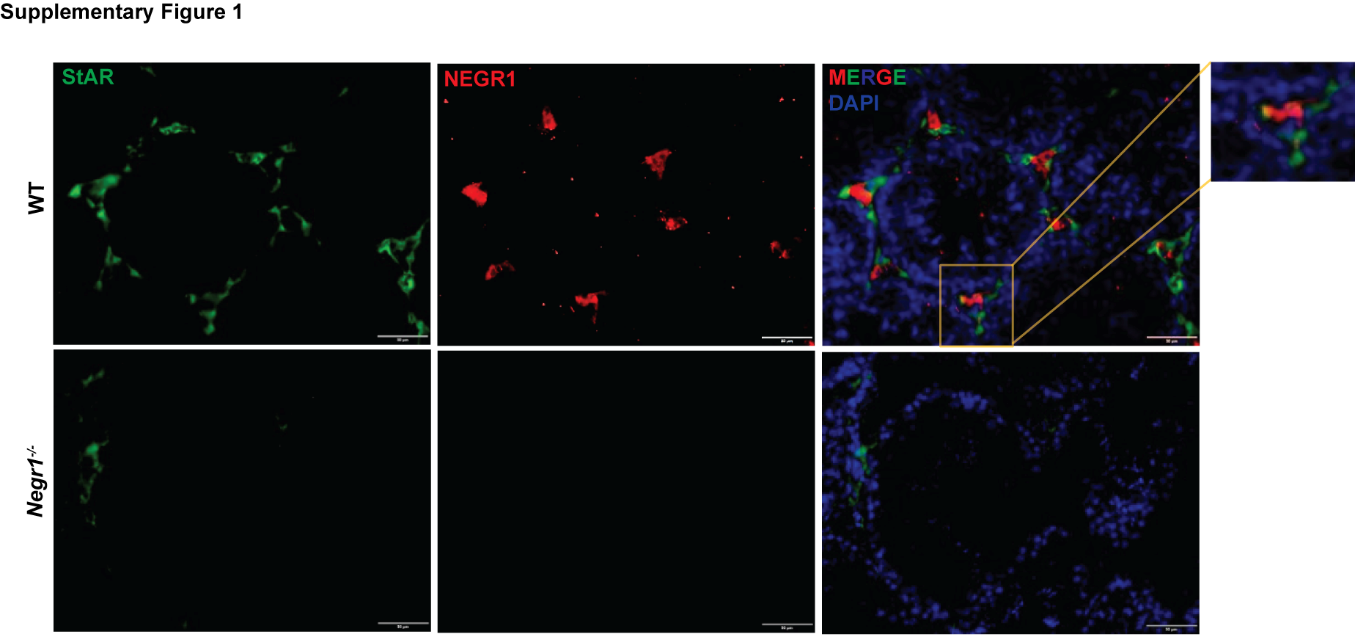
**

**Supplementary Figure S1.** Co-immunofluorescence staining was performed on testis tissue sections from WT and *Negr1^-/-^* mice using anti-NEGR1 and anti-StAR antibodies to assess NEGR1 localization in Leydig cells.

**Supplementary Figure S2**


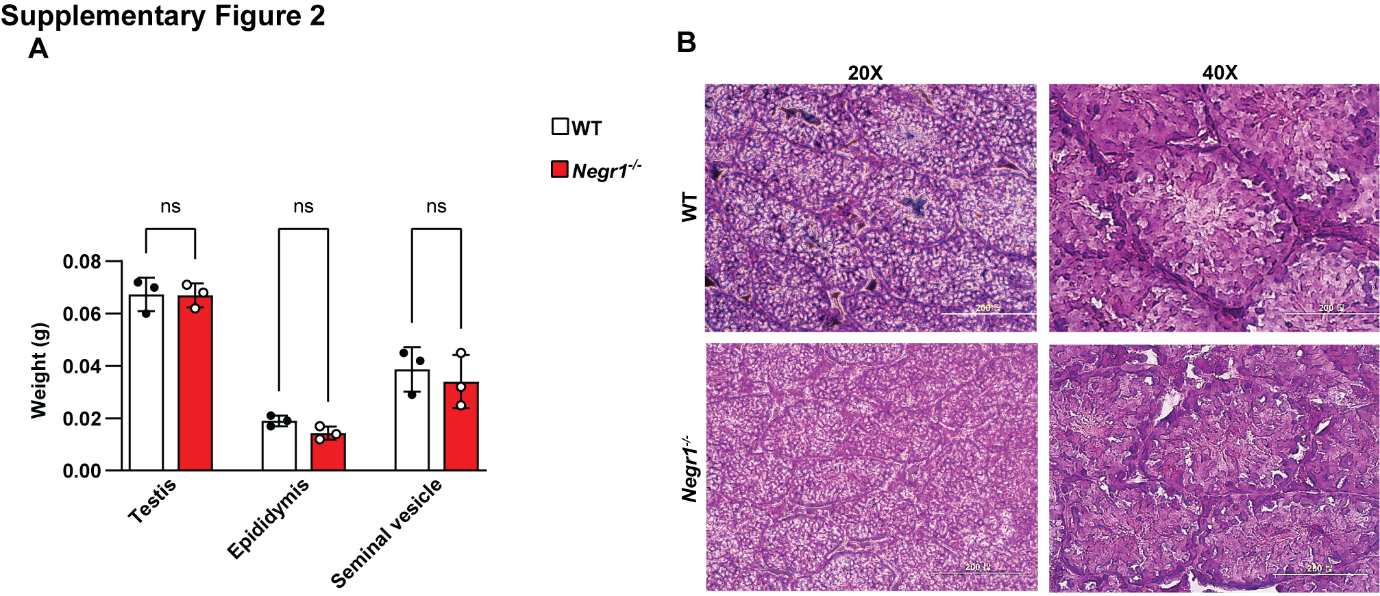


**Supplementary Figure S2.** Analysis of testis tissue of 5-week-old WT and *Negr1^-/-^* mice. (A) The weights of testis, epididymis, and seminal vesicle (n = 3). (B) H&E staining of testicular sections.

**Supplementary Figure S3**

**
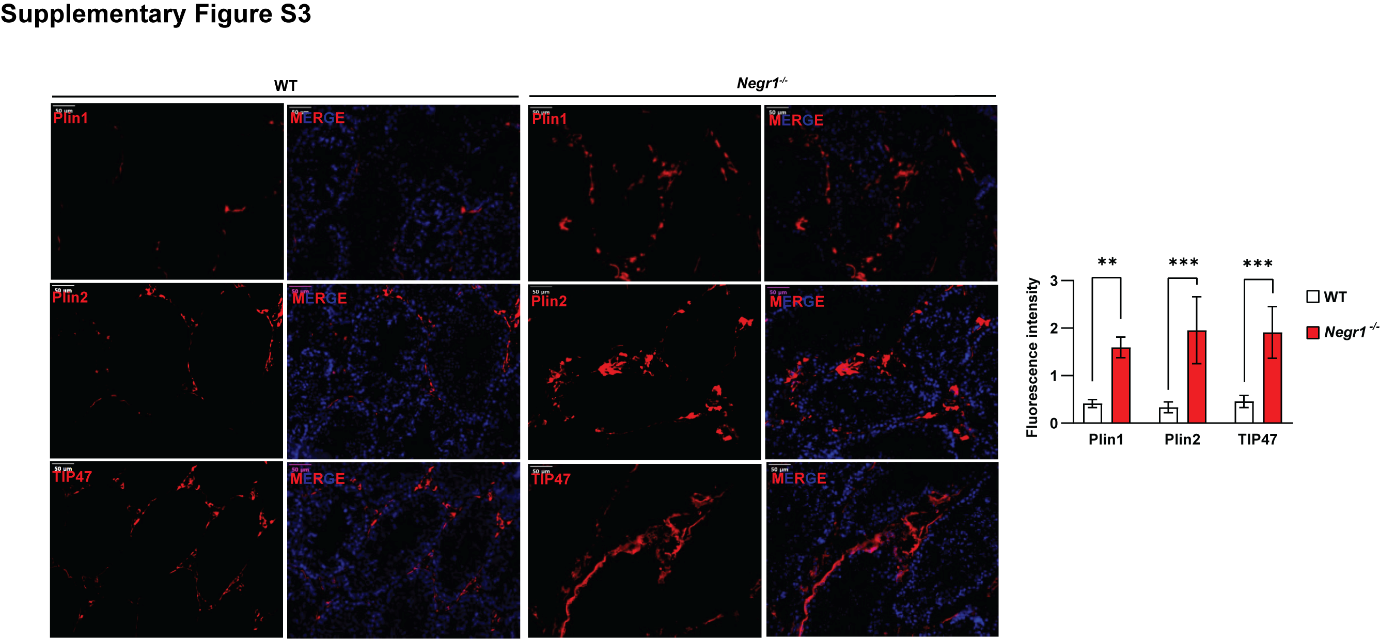
**

**Supplementary Figure S3.** Immunostaining of testicular sections from 12-week-old mice. Plin2, perilipin 2; TIP47, tail-interacting protein of 47 kDa. Data are presented as mean ± SE. ***p* < 0.01, ****p* < 0.001.

**Supplementary Figure S4**

**
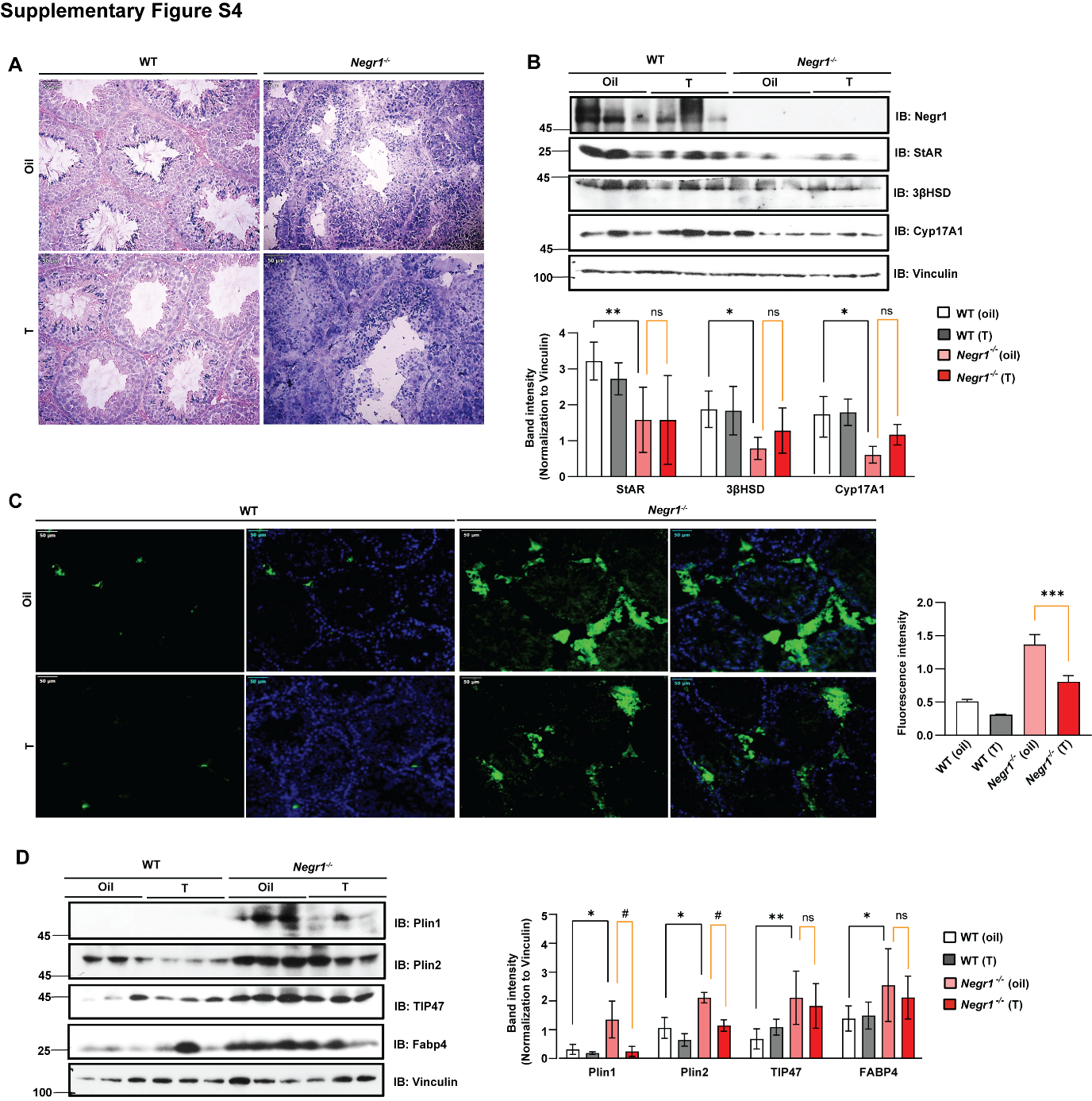
**

**Supplementary Figure S4.** Biochemical and morphological evaluation of testis tissue after testosterone supplementation. WT and *Negr1^-/-^* male mice were injected with vehicle (olive oil) or testosterone (T) for 4 days. (A) H&E staining of testicular sections. (B) Immunoblotting of key steroidogenic enzymes. (C) BODIPY 493/503 staining of testicular sections. (D) Protein expression levels of lipid droplet marker proteins. Data are presented as mean ± SE. ns, not significant. **p* < 0.05, ***p* < 0.01, ****p* < 0.001 versus WT; ^#^*p* < 0.05 versus vehicle (oil). 3βHSD, 3β-hydroxysteroid dehydrogenase; Cyp17A1, cytochrome P450 17A1; StAR, steroidogenic acute regulatory protein; Fabp4, fatty acid binding protein 4.
